# Design and Research of Chromatic Confocal System for Parallel Non-Coaxial Illumination Based on Optical Fiber Bundle

**DOI:** 10.3390/s22249596

**Published:** 2022-12-07

**Authors:** Yali Zhang, Qing Yu, Chong Wang, Yaozu Zhang, Fang Cheng, Yin Wang, Tianliang Lin, Ting Liu, Lin Xi

**Affiliations:** 1 College of Mechanical Engineering and Automation, Huaqiao University, Xiamen 361021, China; 2 Fujian Key Laboratory of Green Intelligent Drive and Transmission for Mobile Machinery, Huaqiao University, Xiamen 361021, China; 3 School of Mechanical Engineering, Anhui Polytechnic University, Wuhu 241000, China

**Keywords:** chromatic confocal system, optical fiber bundle, non-coaxial illumination, transparent specimen, three-dimensional topography reconstruction

## Abstract

Conventional chromatic confocal systems are mostly single-point coaxial illumination systems with a low signal-to-noise ratio, light energy utility and measurement efficiency. To overcome the above shortcomings, we propose a parallel non-coaxial-illumination chromatic-confocal-measurement system based on an optical fiber bundle. Based on the existing single-point non-coaxial-illumination system, the optical fiber bundle is used as the optical beam splitter to achieve parallel measurements. Thus, the system can yield measurements through line scanning, which greatly improves measurement efficiency. To verify the measurement performance of the system, based on the calibration experiment, the system realizes the measurement of the height of the step, the thickness of the transparent specimen and the reconstruction of the three-dimensional topography of the surface of the step and coin. The experimental results show that the measuring range of the system is 200 μm. The measurement accurcy can reach micron level, and the system can realize a good three-dimensional topography reconstruction effect.

## 1. Introduction

With the development of science and technology, three-dimensional topography reconstruction plays an important role in production and life. Chromatic confocal technology is derived from traditional laser confocal technology, which is one of the optical measurement techniques widely used at present. It realizes measurement by using the imaging characteristics of axial dispersion. Additionally, compared with other optical measurement techniques, such as the white-light interferometry [1,2], laser triangle method [3,4], grating projection method [5,6], and laser confocal method [7,8], chromatic confocal technology has no axial scanning, and its measuring efficiency and precision are higher. The application fields of chromatic confocal technology mainly include displacement and thickness measurements [9,10], flatness and roughness tests [11,12], defect and flaw detection [13,14], three-dimensional topography reconstruction [15,16], etc.

In recent years, on the one hand, the research of parallel chromatic confocal technology, which is derived from single-point chromatic confocal technology, is gradually emerging. Similar to laser confocal technology, chromatic confocal technology also uses light-beam splitters [17] to realize parallel measurements. Existing light-beam splitters mainly include the Nipkow turntable [18], micropinhole array [19], microlens array [20], optical fiber bundles [21], the digital micromirror device (DMD) [22]. They all turn single-light spots into multi-light spots to reduce scanning time and improve measurement speed. Among them, the microlens array, DMD and optical fiber bundles have been gradually applied to chromatic confocal parallel-measurement techniques. In 1996, Tiziani H [23] realized multipoint measurement using a microlens array and applied this technology to macro- and microsurface measurements and defect analysis in 2000 [24]. In 2013, Hillenbrand [25] designed a three-point chromatic confocal distance sensor to achieve parallel measurements by two-stage spectral multiplexing. Then, in 2015, the authors of [26] proposed two kinds of chromatic confocal matrix sensors for a three-dimensional-object snapshot collection, which can perform spectral evaluations on all transverse channels at the same time. In 2021, Hao Hu [27] designed a line-scanning chromatic confocal optical path using the slit, which can achieve accurate measurements of three-dimensional topography and the thickness of highly reflective materials. In 2020 and 2021, Yu Qing [28] used optical fiber bundles and the DMD as optical beam splitters to achieve full-field three-dimensional topography measurements of the coaxial-illumination chromatic confocal system. 

On the other hand, with the gradual expansion of application fields, some coaxial-illumination chromatic confocal systems can no longer meet the measurement requirements of specific specimens. Therefore, a non-coaxial-illumination system has gradually emerged in recent years. Non-coaxial illumination occurs when the reflected light and the camera are not on the same axis. In contrast, coaxial illumination occurs when both are on the same axis. The optical path structure of the chromatic confocal system mentioned above is mainly the vertical optical path, which is also called coaxial illumination. With the development of research, chromatic confocal research about non-coaxial-illumination structures is gradually emerging. In 2020, G. Berkovic [29] designed a commercial chromatic-confocal-displacement sensor suitable for the detection of inclined-incident targets. The sensor is fitted with collimation mirrors and reverse reflectors to improve its performance. In 2021, Yu Qing [30] proposed a non-coaxial-illumination-light-path structure to measure the thickness of transparent specimens with micron accuracy.

Based on previous research about parallel chromatic confocal measurements and non-coaxial-illumination structures, the two methods have been combined to improve the measurement efficiency, signal-to-noise ratio and light energy utilization of the system in our paper. Through the usage of an optical fiber bundle with the line-scanning method, the vertical and lateral information of the measured object was captured by the system. Then, the system could reconstruct the three-dimensional topography of the measured surface by the related information.

## 2. Principles of Parallel Chromatic Confocal System with Non-Coaxial Illumination

### 2.1. Principle of Dispersion

**Figure 1:**
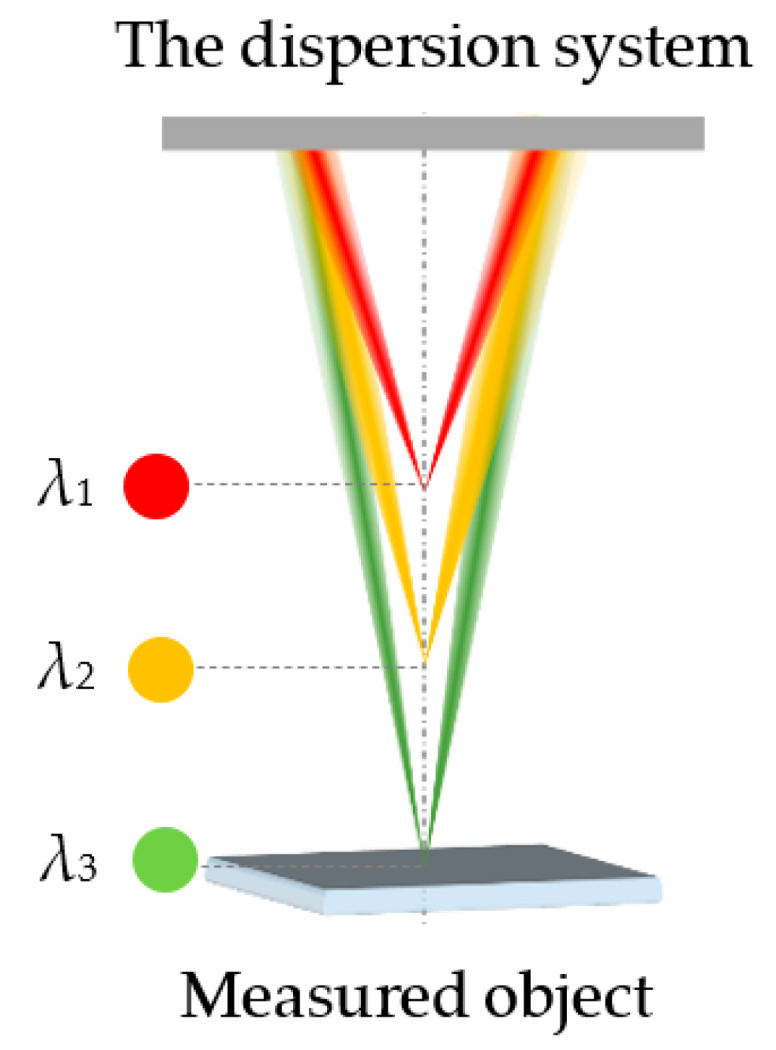
Schematic of the spectral coding technique.

The core principle of the chromatic confocal technique is the spectral coding technique, which focuses the light of different wavelengths onto different axial positions through the dispersion system, as shown in Figure 1. The value of *λ*_1_, *λ*_2_ and *λ*_3_ decrease in turn.

According to the principle of axial dispersion, polychromatic light is dispersed in the direction of the optical axis by the dispersion system. Light of different wavelengths focuses on different axial positions. The wavelength distribution can be expressed as a function of *λ*(*z*), where *z* is the axial position and the output of the light intensity in object space is:

(1) $$I(z)={\left[\frac{\sin(\frac{u}{4})}{\frac{u}{4}}\right]}^{4}$$

In this formula, *u* is the normalized axis coordinate. The normalized axis coordinate *u* can be expressed as:

(2) $$u=\frac{2\pi }{\lambda (z)}\Delta z\frac{a^{2}}{f^{2}}$$

As shown in Equation (2), *λ* is the wavelength of the incident light, *a* is the exit pupil radius of the imaging lens and *f* is the focal length of the lens. Δ*z* is the defocus amount, which is determined by the axial-light-intensity response function of the chromatic-confocal-measurement system. Theoretically, there are a series of intensity peaks on the CCM distribution curve according to *λ*(*z*). This “multi-peak” feature allows CCM systems to perform axial position measurements without mechanical scanning. Thus, the axial position can be measured by decoding the wavelength information of the reflected light.

### 2.2. Principle of Single-Point Chromatic Confocal System with Non-Coaxial Illumination

**Figure 2:**
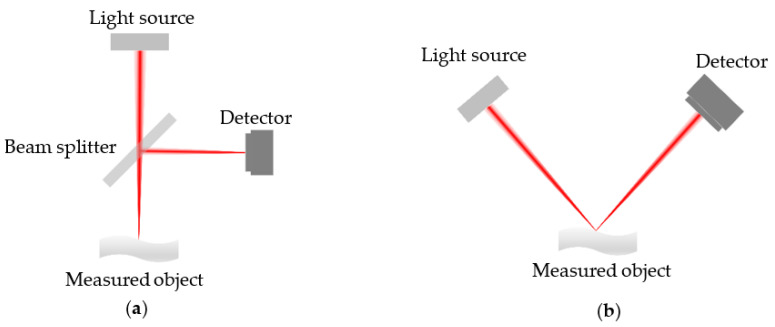
Schematic of the different optical path structures: (**a**) Coaxial illumination; (**b**) Non-coaxial illumination.

Firstly, different optical path structures are shown in Figure 2. The light from the light source travels to the beam splitter, and the light that arrives at the object is reflected from the surface to the detector. When the incident light arrives at the surface at a vertical angle, the optical path structure is called coaxial illumination, as shown in Figure 2a. On the other hand, when the incident light arrives at the surface at an inclined angle, the optical path structure is called non-coaxial illumination, as shown in Figure 2b.

**Figure 3:**
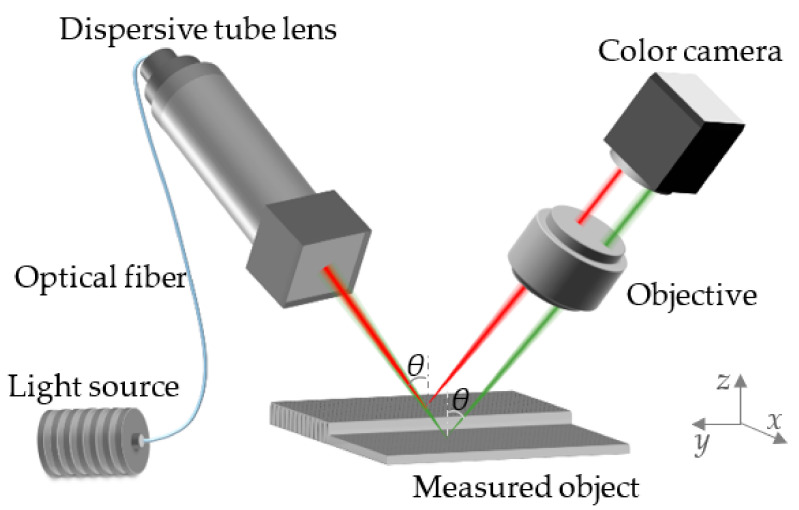
Schematic of the single-point chromatic confocal system with non-coaxial illumination.

Secondly, the schematic of the single-point chromatic confocal system with non-coaxial illumination is shown in Figure 3, where *θ* represents the angle of the incident light. The light beams with different wavelengths emitted from the white-light source are distributed along the optical axis by the dispersive tube lens and are then focused on different axial locations. In this process, only the light beams focused on the surface of the sample can be reflected and focused by the objective. Finally, the light spots with corresponding color information are captured by the imaging surface of the color camera. Combined with the different color information corresponding to the different heights of the object surface, the three-dimensional topography information can be obtained by the two-dimensional point-scanning method.

The point spread function (PSF) describes the imaging response of the point light source, and it is often used to measure the resolution of reconstructed images.

When the point light source in the single-point system arrives at the surface of the measured object, the PSF function *h*_0_ of the system is:

(3) $$\begin{array}{l} h0(x,y,z)\\ =\int \delta (x,y,z-\Delta z)\cdot h_{1}(x,y,z)dz\\ =\delta (x,y)h_{1}(x,y,\Delta z) \end{array}$$

In Equation (3), Δ*z* represents the longitudinal defocus quantity. Then, the light spot that arrives at the surface is reflected by the measured surface and arrives at the imaging plane. So, the PSF function of the whole single-point system is:

(4) $$\begin{array}{l} h_{conf}(x,y,z)=\delta (x,y)h1(x,y,\Delta z)*h2(x,y,\Delta z)\\ =\int \int \delta (x{}^{\prime },y{}^{\prime })h1(x{}^{\prime },y{}^{\prime },\Delta z)\cdot h2(x-x{}^{\prime },y-y{}^{\prime },\Delta z)dx{}^{\prime }dy{}^{\prime }\\ =h1(0,0,\Delta z)h2(x,y,\Delta z) \end{array}$$

### 2.3. Principle of the Optical Fiber Bundle

**Figure 4:**
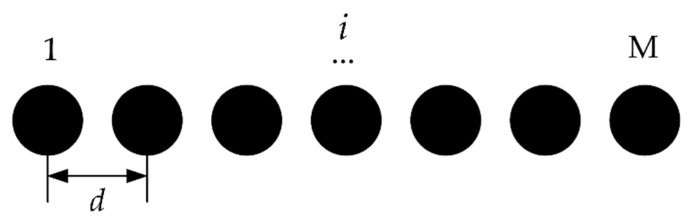
The point source of linear array.

The optical fiber bundle, also known as the optical fiber image-transmission bundle, is composed of several optical fibers. It has several array arrangements, including circular, square and linear arrays. In this paper, the light emitted from the optical fiber bundle is linear, as shown in Figure 4. In Figure 4, the number of the optical fibers is represented by M, and the center distance between the adjacent light spots is represented by *d*.

It can be seen from Equation (4) that in parallel measurements, the imaging rule of any point light source satisfies the confocal relation of the impulse response function. Then, the amplitude distribution of the light field of the light source is:

(5) $$G(x0,y0,z0)=\sum_{i=1}^{M}\delta 2(x0-id,y0)$$

In Equation (5), *δ*_2_ represents the two-dimensional *δ* function and *d* represents the distance between the adjacent point light sources. Therefore, the PSF function of the whole parallel system is:

(6) $$\begin{array}{l} h_{conf}=\left[\sum_{i=1}^{M}\delta 2(x0-id,y0)\cdot h1(x-id,y,\Delta z)\right]*h2(x,y,\Delta z)\\ =\sum_{i=1}^{M}\int \int \delta 2(x{}^{\prime }-id,y{}^{\prime })h1(x{}^{\prime }-id,y{}^{\prime },\Delta z)\cdot h2(x-x{}^{\prime },y-y{}^{\prime },\Delta z)dx{}^{\prime }dy{}^{\prime }\\ =\sum_{i=1}^{M}h1(0,0,\Delta z)h2(x-id,y,\Delta z) \end{array}$$

From Equation (6), in the parallel chromatic confocal measurement system, the optical field distribution is a total of each single-point system. It is equivalent to the coefficient of M single-point chromatic confocal subsystems. The above theoretical analysis provides a theoretical basis for the application of optical fiber bundle in the parallel chromatic confocal system. 

**Figure 5:**
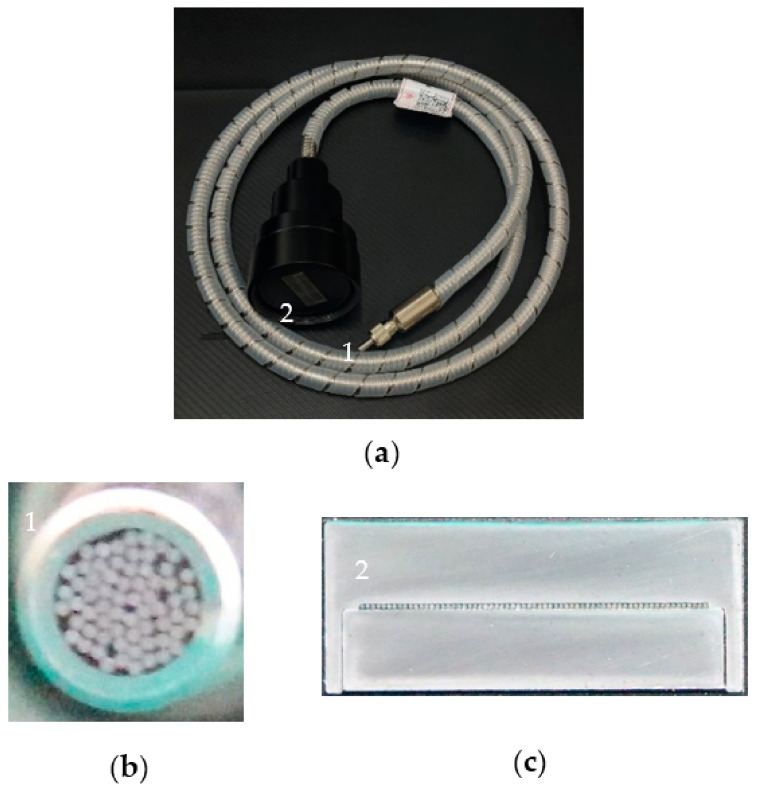
(**a**) The physical picture of the optical fiber bundle; (**b**) Circular end; (**c**) Linear end.

Under the measurement conditions, the optical fiber bundle applied in this system is shown in Figure 5a. The section of the end coupled with the light source is circular, as shown in Figure 5b. The section of the end coupled with the dispersion tube lens is linear, as shown in Figure 5c. In Figure 5, the number “1” represents the circular end, and the number “2” represents the linear end. 

**Figure 6:**
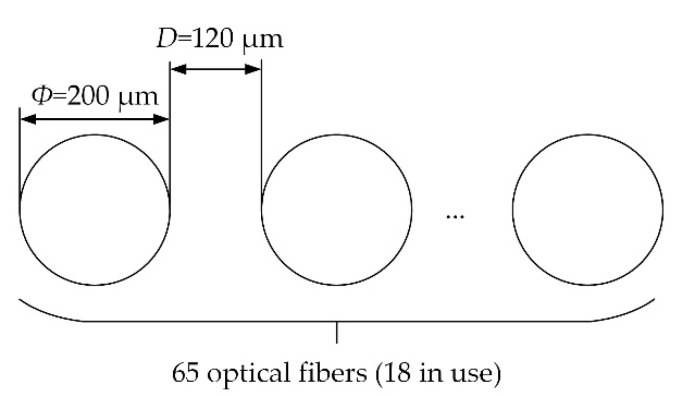
Parameter diagram of the optical fiber bundle.

From Figure 5b,c, it can be seen that the optical fiber bundle used in our system is composed of 65 multi-mode fibers, and the material of the fiber core is quartz. The parameter diagram of the optical fiber bundle is shown in Figure 6. The parameters of each optical fiber are as follows: the NA value is 0.22; the diameter is 200 μm; the distance *D* between fibers is 120 μm; and the diameter of the circular end is about 2 mm. Due to the limitation of the aperture of the components, only 18 of the 64 optical fibers are applied in our system. Therefore, the actual length of the optical fiber bundle array used in the system is about 5.66 mm.

### 2.4. Principle of Parallel Chromatic Confocal System with Non-Coaxial Illumination

**Figure 7:**
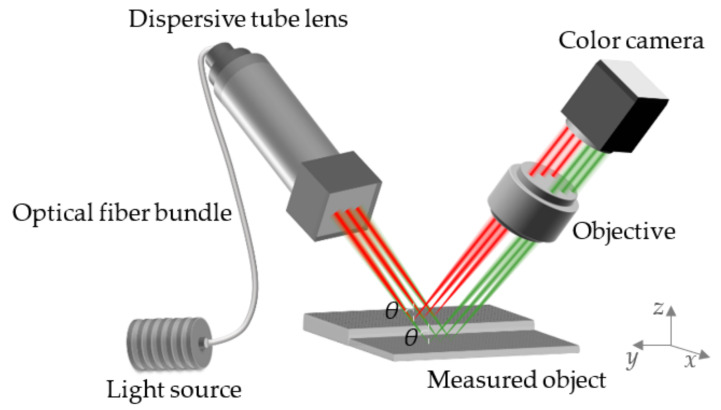
Schematic of the parallel chromatic confocal system with non-coaxial illumination.

After introducing the principle of the optical fiber bundle, we apply it to Section 2.2 to obtain the parallel chromatic confocal system with non-coaxial illumination based on the optical fiber bundle, as shown in Figure 7, where *θ* represents the inclined angle of the incident light. The light generated from the optical fiber bundle is arranged linearly on the measured surface. When the measured object moves along the direction (the *x*-axis in Figure 7) that is perpendicular to the optical fiber bundle and the *z*-axis of the system, the measurement can be completed through line scanning. In this process, the three-dimensional topography of the whole measured object can be obtained.

### 2.5. Principle of the Color-Conversion Algorithm

**Figure 8:**
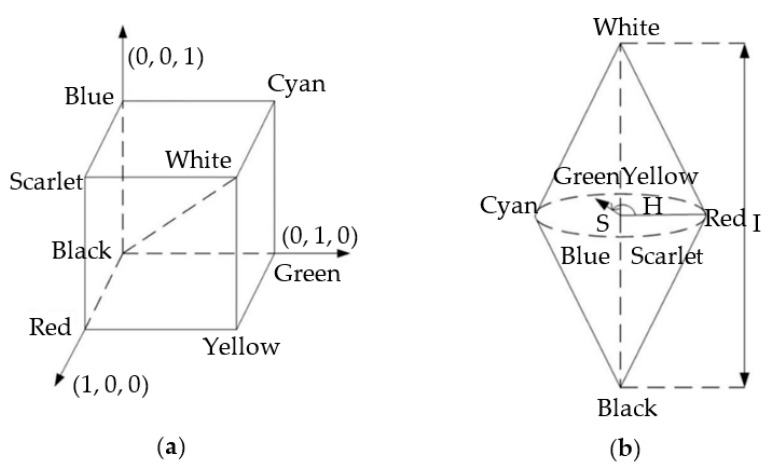
(**a**) Model of the RGB color space; (**b**) Model of the HSI color space.

In our study, a color-conversion algorithm is developed, which mainly involves the RGB color space and HSI color space, and the two color-space models are shown in Figure 8.

The HSI color model starts from the human visual system. The three related parameters used to describe colors are H (hue), S (saturation) and I (intensity). The H value, which is a wavelength-dependent parameter, can describe the spectral color and its range is [0, 2π]. Each spectral color has one angle for itself. For example, the angle of spectral red is 0, the angle of spectral green is 2π/3 and the angle of spectral blue is 4π/3. 

Because the original data from the color camera is in RGB format, a conversion algorithm for RGB to HSI is needed. The classic geometric derivation conversion formula for the conversion is given as follows:

(7) $$H=\left\{\begin{array}{c} \theta ,\\ 2\pi -\theta , \end{array}\begin{array}{c} G\geq B\\ G<B \end{array}\right.$$

where $\theta =\cos^{-1}\left(\frac{\left(R-G)+(R-B\right)}{2\sqrt{{\left(R-G\right)}^{2}+(R-B)(G-B)}}\right)$.

In the following section, the relevant experiments are discussed to verify the principles discussed above.

## 3. Experiments

### 3.1. Construction of the Measurement System

**Figure 9:**
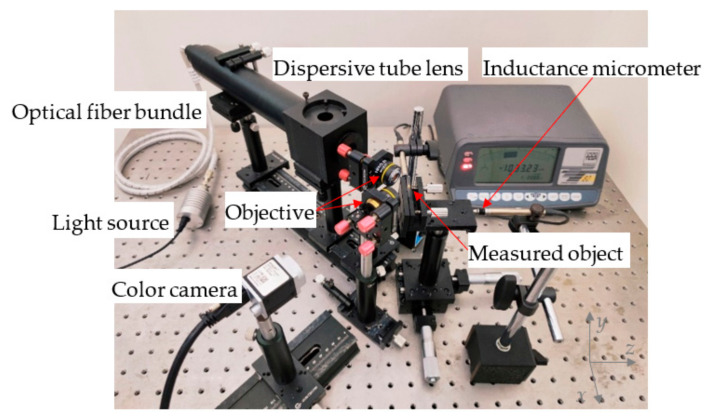
Device diagram of the parallel chromatic confocal system with non-coaxial illumination.

Based on the above theoretical analysis, the parallel chromatic confocal system with a non-coaxial-illumination-measurement experimental platform based on an optical fiber bundle is established, as shown in Figure 9. The components used in the experimental platform are listed in Table 1.

### 3.2. Calibration Experiment

**Figure 10:**
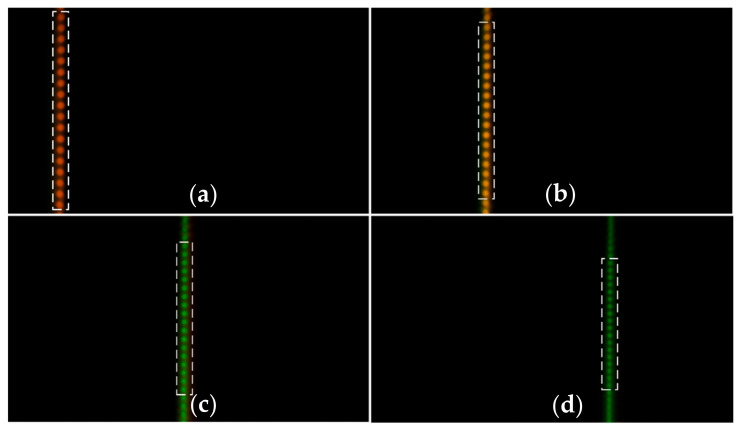
Images at different axial positions obtained by the camera: (**a**): 50 μm; (**b**): 350 μm; (**c**): 650 μm; (**d**): 950 μm.

First of all, the calibration experiment is conducted. After the experimental platform is built, a standard measuring block is selected as the measured object. The platform is controlled by the inductance micrometer to perform line scanning along the *z*-direction at a fixed step of 50 μm. In the calibration process, the images at different *z*-axial positions of 50 μm, 350 μm, 650 μm and 950 μm are obtained by the color camera, as shown in Figure 10.

We use the independently developed color-conversion algorithm mentioned in Section 2.5 to process the obtained color images. According to the color information of each light spot and the corresponding displacement information, the “H value–Axial displacement” calibration curve is obtained by calculating the average H value of 18 optical fibers of spots selected by the dotted box in Figure 10. The calculation results are shown in Table 2.

**Figure 11:**
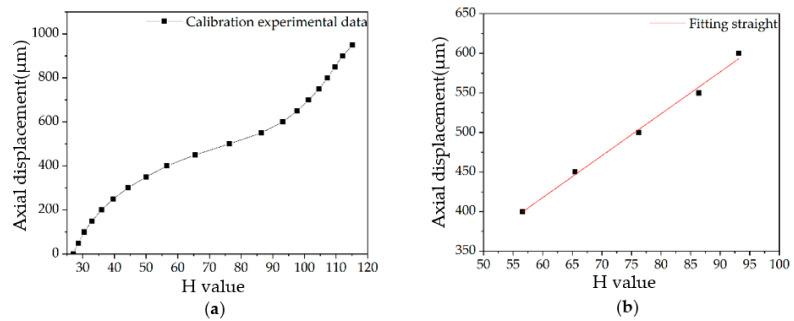
Calibration experiment: (**a**) Experimental data; (**b**) Calibration fitting results.

The calibration results are shown in Figure 11a. In the calibration curve, the linear section is the measurement range of the system, which is 200 μm. Through the linear fitting of the calibration data within the linear measurement range, the fitting line is obtained as shown in Figure 11b.

From Figure 11b, the calibration equation of the system is:

(8) y1=5.29x+200.23

where *y*_1_ is the axial displacement value of the measured object, the unit is μm and *x* represents the corresponding H value. According to the calibration experiment, the measuring range of the system is 200 μm. The linear correlation coefficient of the calibration equation is above 0.99.

### 3.3. Measurement of Step Height

**Figure 12:**
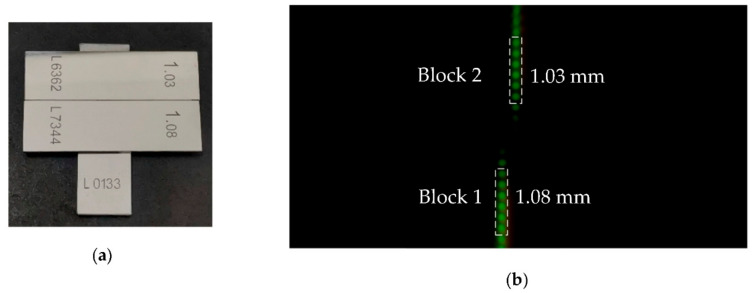
Measurement of step height: (**a**) Picture of step; (**b**) Schematic diagram of step measurement.

Based on the calibration experiment, the step measurement experiment is carried out. In the experiment, two standard measuring blocks with a height of 1.03 mm and 1.08 mm are selected. The block of 1.08 mm is Block 1, and the block of 1.03 mm is Block 2. They are ground together on the base by molecular force to form step, and the base block gauge is random. The step is shown in Figure 12a. 

The true height value of the step measured by an inductance micrometer Tesa TT80 is 57.87 μm. The surface of the step is within the linear measurement range of the system and the schematic diagram is shown in Figure 12b. 

**Figure 13:**
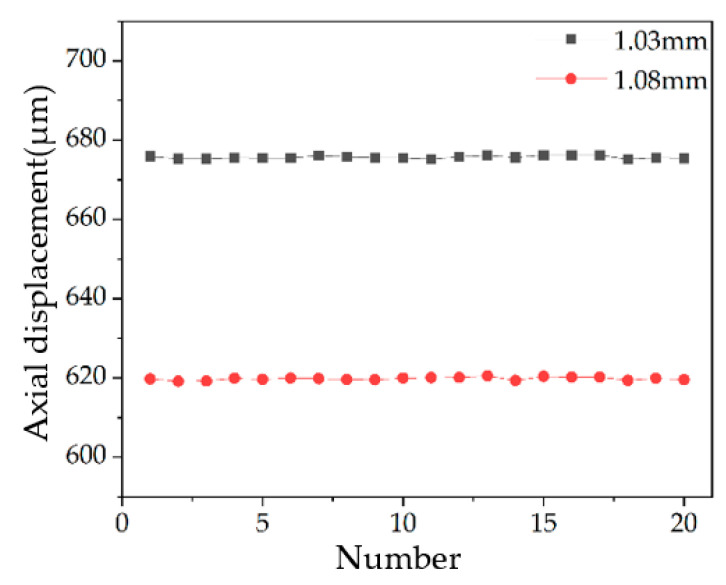
Result diagram of step measurement.

In Figure 12b, the six light spots fall on the measuring block surface of 1.03 mm and 1.08 mm, respectively. By calculating the average H value of the six light spots of Block 1 and Block 2, the height values and the difference in the height of the step of the two measuring blocks can be obtained. The diagram of measurement results is shown in Figure 13, and the above experimental process was repeated 20 times. The results are shown in Table 3.

Through the analysis of the above data, it can be seen that the measured height of the step is 55.84 μm, the relative error is −3.51% and the 3σ value is 0.87 μm. Therefore, the measurement accuracy of the system can reach the micron level.

### 3.4. Measurement of Transparent Specimen Thickness

**Figure 14:**
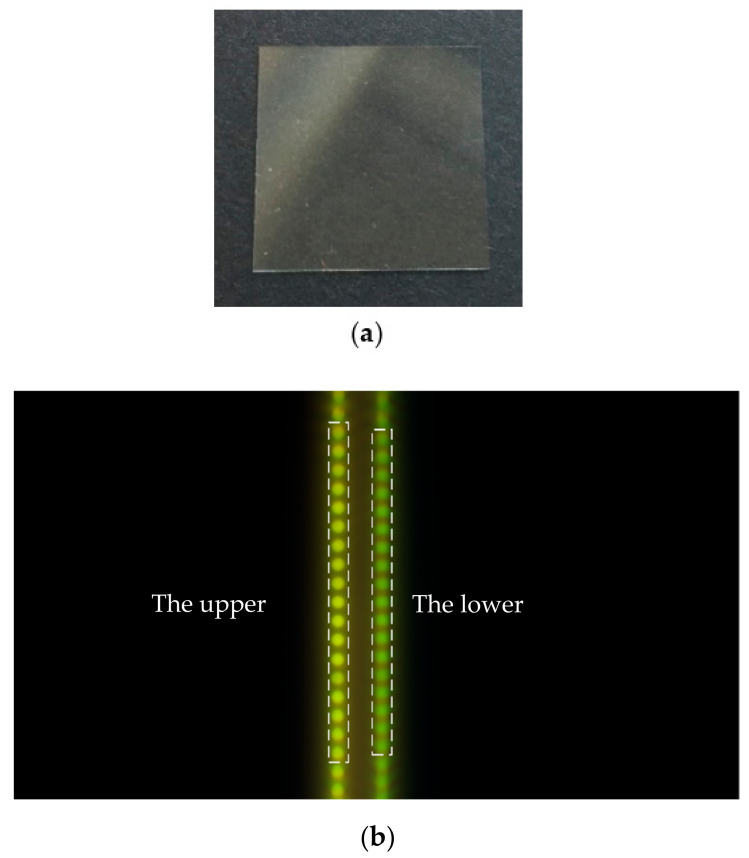
Measurement of transparent specimen thickness: (**a**) Picture of the transparent specimen; (**b**) Schematic of the measurement of the transparent specimen.

In order to verify the ability of the system to measure the thickness of transparent specimens, a glass slide with the transmittance of 84% (380 nm)–90% (717 nm) is selected as the transparent specimen for measurement. The true value of the glass slide measured by Tesa TT80 is 184.08 μm. The physical image of the glass slide is shown in Figure 14a, and the schematic diagram is shown in Figure 14b. In Figure 14b, the 18 light spots on the left are the H value reflected on the upper surface of the transparent specimen, and the 18 light spots on the right are the H value reflected on the lower surface of the transparent specimen.

Similarly, 20 groups of repetitive measurement experiments are conducted based on the calibration experiment. Additionally, the thickness of the transparent specimen *d* can be expressed as follows [30]:

(9) $$d=k_{1}(H_{i}-H_{0})\cdot \cos\theta \cdot \frac{\left(\tan\alpha -\tan\beta \right)}{\left(\tan\alpha {}^{\prime }-\tan\beta {}^{\prime }\right)}$$

**Figure 15:**
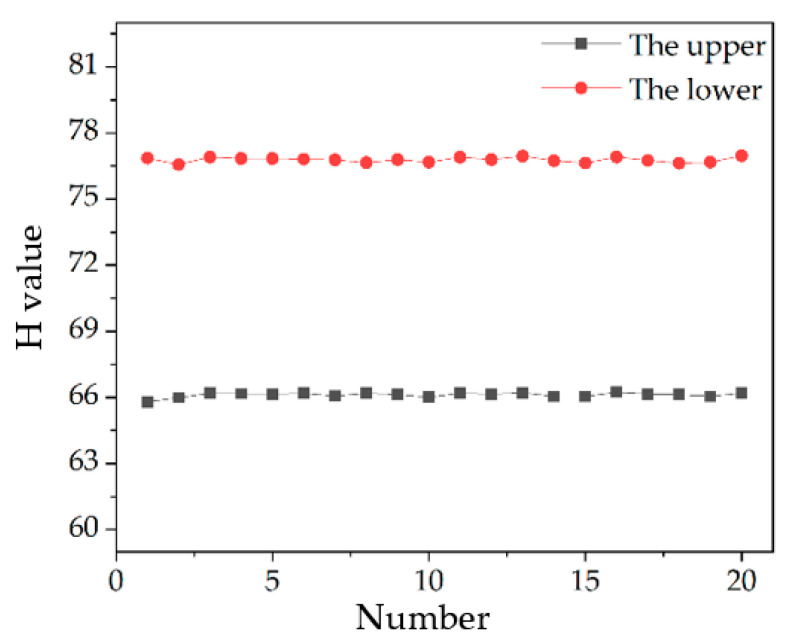
Diagram of measurement results of transparent specimen.

The thickness data of transparent specimen obtained is shown in Figure 15 and Table 4.

The measured thickness of the transparent specimen is 177.58 μm, the relative error is −3.53% and the 3σ value is 0.36 μm. The measurement accuracy of the system can reach the micron level.

### 3.5. Three-Dimensional Topography by Line-Scanning Method

**Figure 16:**
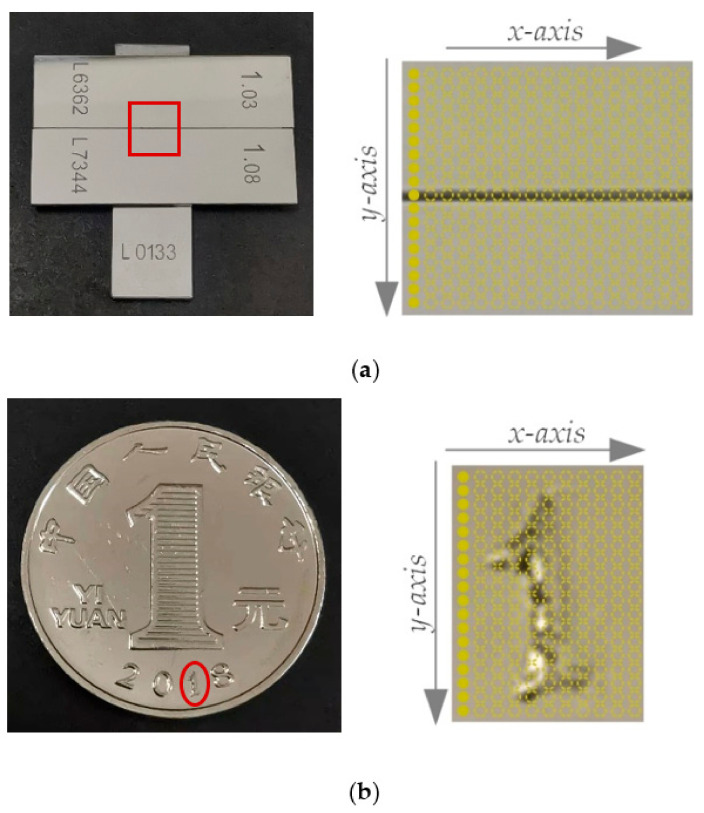
Three-dimensional topography by the line-scanning method: (**a**) Schematic of the step measurement; (**b**) Schematic of the coin measurement.

In order to verify the ability of three-dimensional surface topography, the step in Section 3.3 and the “1” character on the surface of the coin were chosen as measured objects. The physical picture and the schematic diagram of the step and CNY 1 coin are shown in Figure 16.

**Figure 17:**
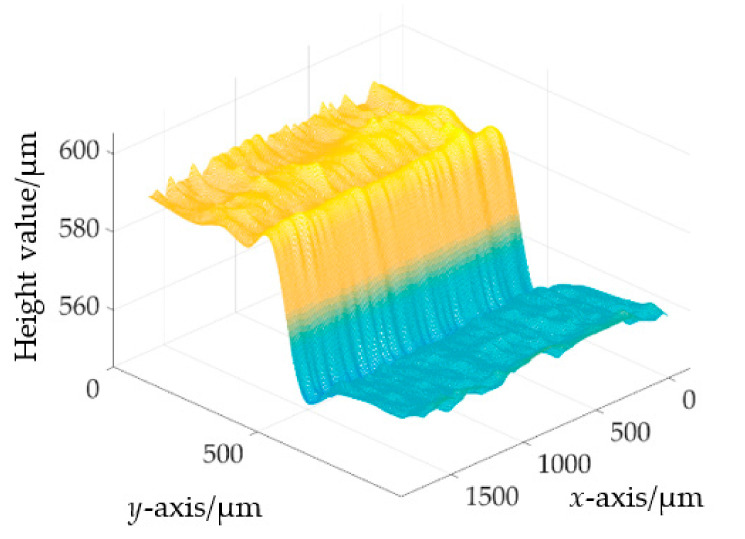
The three-dimensional diagram of the step.

For the restoration of the step surface, the optical fiber bundle is arranged in the *y*-axis direction as shown in Figure 16a, and the motor moves in the *x*-axis direction. In the actual measurement process, the measuring range of the step surface is 3.25 mm × 5.92 mm. The one-dimensional scanning distance is 3.25 mm, and the scanning speed is 50 μm/s. The photography speed of the color camera is 1 piece/s and a total of 66 photos are taken. Each photo has 12 measuring points, so there is a total of 792 measuring points. Through the “H value-displacement” conversion processing of 792 measured points, the three-dimensional topography features of the step surface are obtained, as shown in Figure 17 in which the surface height information of the step surface is about 50 μm.

**Figure 18:**
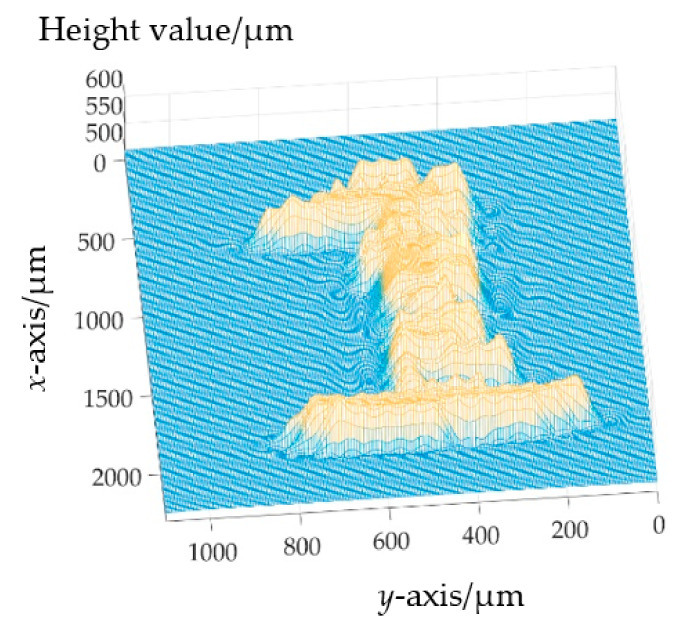
The three-dimensional diagram of “1”.

For the restoration of the “1” character on the surface of the coin, the optical fiber bundle is arranged in the *y*-axis direction as shown in Figure 16b, and the motor moves in the *x*-axis direction. In the actual measurement process, the “1” character measuring range is 2.20 mm × 1.02 mm. The one-dimensional scanning distance is 2.20 mm, and the scanning speed is 25 μm/s. The color camera photography speed is 1 piece/s and a total of 89 photos are taken. Each photo has 18 measuring points with a total of 1602 measuring points. By data analysis and the processing of these measuring points, the three-dimensional topography feature of “1” is obtained, as shown in Figure 18 in which the measured surface height is about 50 μm.

### 3.6. Contrast Experiment

In order to better verify the improvement of the measurement efficiency of the system in this paper, compared with the single-point non-coaxial-illumination system, the corresponding contrast experiments are conducted.

**Figure 19:**
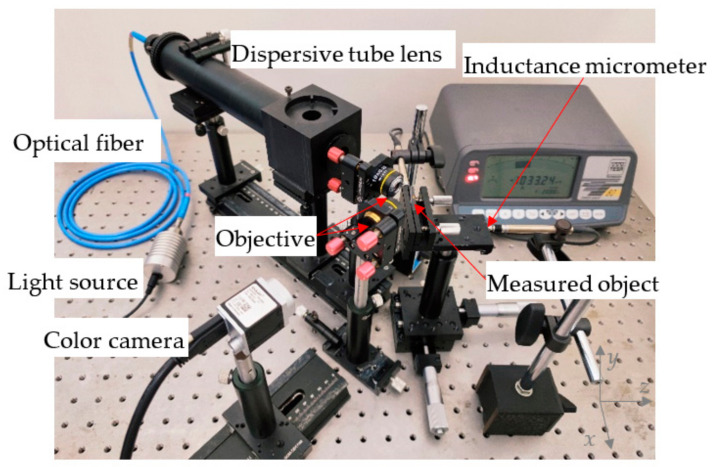
Device diagram of the single-point chromatic confocal system with non-coaxial illumination.

The experimental platform of the single-point chromatic confocal system with non-coaxial illumination is shown in Figure 19.

**Figure 20:**
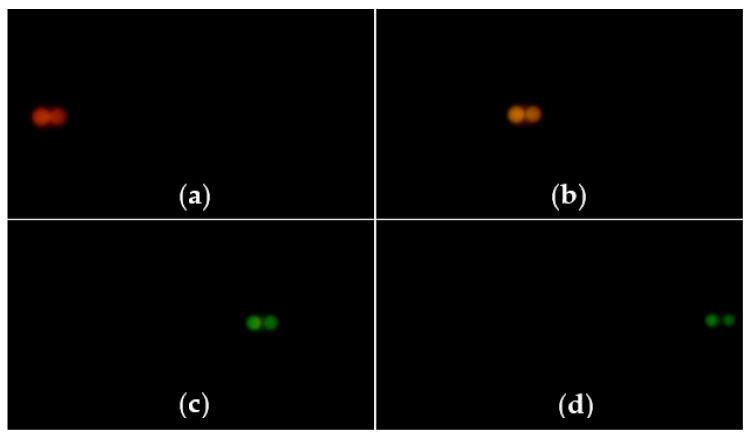
Images at different axial positions obtained by the camera: (**a**): 100 μm; (**b**): 700 μm; (**c**): 1300 μm; (**d**): 1900 μm.

In the above system, under the condition that the other experimental conditions remain unchanged, the optical fiber bundle is replaced with an optical fiber. According to the above-mentioned experimental procedures, the calibration experiment and the transparent specimen-thickness-measurement experiment are carried out. In the calibration process, the images at different *z*-axial positions of 100 μm, 700 μm, 1300 μm and 1900 μm obtained by the color camera are shown in Figure 20.

As can be seen from the number of light spots in the calibration results between Figure 10 and Figure 20, the measurement efficiency of the parallel measurement is 18 times that of the single-point measurement. The total experimental results of the parallel measurement are presented in Table 5.

From Table 5, it can be found that the measurement accuracy of the two systems can reach the micron level regardless of whether the optical fiber bundle is used as the beam splitter or not. Because the number of light spots in the optical fiber bundle in the parallel non-coaxial-illumination system is 18 times that in the single-point non-coaxial-illumination system, the measurement efficiency of the parallel non-coaxial-lighting system is significantly improved.

## 4. Discussion

This paper proposes a chromatic confocal system for parallel non-coaxial illumination based on the optical fiber bundle, and it solves the problem of a low signal-to-noise ratio and low light energy utility which are in the traditional coaxial illumination system. This paper includes the following points:

(1) By the related theoretical analysis of the non-coaxial-illumination system and the properties of the optical fiber bundle, it is proved that the optical fiber bundle can be applied in the chromatic confocal system. Based on this, the existing single-point non-coaxial-illumination system is optimized using the optical fiber bundle as the light-beam splitter to realize parallel measurements, and this optimization can improve the measurement efficiency of the single-point system.
(2) Combined with the color-conversion algorithm, the conclusion of (1) is verified by corresponding step height measurement, transparent specimen thickness measurement and three-dimensional topography restoration. Three-dimensional topography includes the restoration of the step and character “1” of the coin.
(3) The experimental results show that the measuring range of the system is 200 μm, the repeatability is better than 0.87 μm, the relative error is less than ±4% and the measurement accuracy can reach micron level. Additionally, the measurement efficiency of the proposed system is 18 times higher than that of the single-point non-coaxial-illumination system.

## 5. Conclusions

In this paper, we propose a chromatic confocal system for parallel non-coaxial illumination. Based on the optical fiber bundle and the existing single-point non-coaxial-illumination system, the optical fiber bundle as a light-beam splitter achieves parallel measurements through line scanning, which greatly improves the measurement efficiency of the single-point non-coaxial-illumination system. The experimental results show that the measurement accuracy can reach micron level and the system has a good three-dimensional topography reconstruction effect. In the future, we will further improve the measurement accuracy of the system in three-dimensional topography. Additionally, efforts will also be made to develop the system towards miniaturization and integration.

## Figures and Tables

**Table 1:** List of components used in our system.

Components	Manufacturer	Function
White-light source	Yousheng, (MT-G2 Easy White LED)	Produce polychromatic light source
Optical fiber bundle	Yousheng, (Custom-made)	Divide a light beam into several beams
Dispersive tube lens	Self-built	Produce chromatic dispersion
Objective	Motic, (Magnification:10×; N.A value:0.1)	Focus on the light
Platform	Daheng Optics, (GCM-T25MC)	Adjust and provide displacement
Gauge block	WD, (32 pieces of level 0)	As the measured object
Transparent specimen	Sail brand	As the measured object
Inductance micrometer	Tesa, (TT80)	Measure displacement value and true value
Color camera	Basler, (a2A5320-23ucBAS)	Capture color images

**Table 2:** Calibration experimental data.

Number	Axial Displacement (μm)	H Value
1	0	26.93
2	50	28.58
3	100	30.46
4	150	32.88
5	200	35.89
6	250	39.57
7	300	44.24
8	350	49.94
9	400	56.56
10	450	65.47
11	500	76.23
12	550	86.36
13	600	93.12
14	650	97.70
15	700	101.27
16	750	104.63
17	800	107.28
18	850	109.58
19	900	112.03
20	950	115.20

**Table 3:** Step measurement experiment data.

Number	H Value of Block 1	Displacement of Block 1 (μm)	H Value of Block 2	Displacement of Block 2 (μm)	Height of Difference (μm)
1	89.91	675.85	79.31	619.78	56.07
2	89.80	675.27	79.20	619.20	56.07
3	89.80	675.27	79.21	619.25	56.02
4	89.85	675.54	79.34	619.94	55.60
5	89.84	675.48	79.28	619.62	55.86
6	89.83	675.43	79.35	619.99	55.44
7	89.96	676.12	79.32	619.83	56.29
8	89.89	675.75	79.28	619.62	56.13
9	89.85	675.54	79.26	619.52	56.02
10	89.84	675.48	79.34	619.94	55.54
11	89.79	675.22	79.37	620.10	55.12
12	89.89	675.75	79.37	620.10	55.65
13	89.96	676.12	79.45	620.52	55.60
14	89.87	675.64	79.23	619.36	56.28
15	89.98	676.22	79.43	620.41	55.81
16	89.97	676.17	79.39	620.20	55.97
17	89.98	676.22	79.40	620.26	55.96
18	89.78	675.17	79.24	619.41	55.76
19	89.86	675.59	79.33	619.89	55.70
20	89.83	675.43	79.27	619.57	55.86
The average value of the step (μm)					55.84
The difference from the true value (μm)					−2.03
Relative error					−3.51%
The standard deviation σ of the step (μm)					0.29

**Table 4:** Transparent specimen thickness measurement experimental data.

Number	H Value of Upper	H Value of Lower	H Value of the Difference
1	65.80	76.86	11.06
2	65.99	76.57	10.58
3	66.19	76.91	10.72
4	66.17	76.84	10.67
5	66.15	76.84	10.69
6	66.19	76.81	10.62
7	66.06	76.78	10.72
8	66.19	76.65	10.46
9	66.14	76.79	10.65
10	66.00	76.68	10.68
11	66.20	76.90	10.70
12	66.15	76.79	10.64
13	66.21	76.95	10.74
14	66.05	76.74	10.69
15	66.05	76.64	10.59
16	66.23	76.91	10.68
17	66.16	76.75	10.59
18	66.13	76.63	10.50
19	66.05	76.68	10.63
20	66.20	76.97	10.77
The average H value of the difference			10.67
The thickness value of the transparent specimen(μm)			177.58
The difference from the true value(μm)			−6.5
Relative error			−3.53%
The standard deviation σ of the difference(μm)			0.12

**Table 5:** Experimental comparison results.

Experiment Type	Experiment Results	Our System	The Comparative System
Calibration experiment	Calibration equation	y1=5.29x+200.23	y2=12.70x+335.12
	Measuring range (μm)	200	400
	Linear correlation coefficient	>0.99	>0.99
Measurement of transparent specimen thickness	Measured thickness value (μm)	177.58	176.00
	Relative error	−3.53%	−4.39%
	Standard deviation *σ* (μm)	0.12	0.01

## Data Availability

Data sharing is not applicable to this article.
